# Should multiple imputation be stratified by exposure group when estimating causal effects via outcome regression in observational studies?

**DOI:** 10.1186/s12874-023-01843-6

**Published:** 2023-02-16

**Authors:** Jiaxin Zhang, S Ghazaleh Dashti, John B. Carlin, Katherine J. Lee, Margarita Moreno-Betancur

**Affiliations:** 1 Clinical Epidemiology and Biostatistics Unit, Department of Paediatrics, University of Melbourne, Parkville, Australia; 2 Clinical Epidemiology and Biostatistics Unit, Murdoch Children’s Research Institute, 50 Flemington Road, 3052 Parkville, Australia

**Keywords:** Causal inference, Multiple imputation, Outcome regression, Observational study, Missing data, Target trial

## Abstract

**Background:**

Despite recent advances in causal inference methods, outcome regression remains the most widely used approach for estimating causal effects in epidemiological studies with a single-point exposure and outcome. Missing data are common in these studies, and complete-case analysis (CCA) and multiple imputation (MI) are two frequently used methods for handling them. In randomised controlled trials (RCTs), it has been shown that MI should be conducted separately by treatment group. In observational studies, causal inference is now understood as the task of emulating an RCT, which raises the question of whether MI should be conducted by exposure group in such studies.

**Methods:**

We addressed this question by evaluating the performance of seven methods for handling missing data when estimating causal effects with outcome regression. We conducted an extensive simulation study based on an illustrative case study from the Victorian Adolescent Health Cohort Study, assessing a range of scenarios, including seven outcome generation models with exposure-confounder interactions of differing strength.

**Results:**

The simulation results showed that MI by exposure group led to the least bias when the size of the smallest exposure group was relatively large, followed by MI approaches that included the exposure-confounder interactions.

**Conclusions:**

The findings from our simulation study, which was designed based on a real case study, suggest that current practice for the conduct of MI in causal inference may need to shift to stratifying by exposure group where feasible, or otherwise including exposure-confounder interactions in the imputation model.

**Supplementary Information:**

The online version contains supplementary material available at 10.1186/s12874-023-01843-6.

## Introduction

Causal inference is a common objective in epidemiological research. Various methodological developments in the past few decades aim to aid researchers in answering causal questions from observational studies. Hernán and co-authors have proposed a framework for conceptualising the usual causal estimand, the average causal effect (ACE), through the specification of the hypothetical randomised controlled trial (RCT) one would have liked to conduct, the so-called “target trial” [1]. This estimand is estimable in observational studies under a set of identifiability assumptions [2, 3]. Although more sophisticated methods for estimating the ACE are available, most researchers still routinely use outcome regression, especially in the simple single-point exposure and outcome setting. This approach assumes that the ACE is constant across the confounder strata (i.e. there is no effect modification) [4].

The analysis of observational studies is often complicated by missing data [5]. One easy but potentially problematic method for handling missing data is complete-case analysis (CCA), which only uses records with complete observations in the target analysis [6–8]. To overcome some of the limitations of CCA, such as potential bias and loss of precision, more principled methods have been proposed in recent decades, in particular, multiple imputation (MI) [9–11]. MI is a two-stage approach, which firstly creates multiple complete datasets by imputing missing values from approximate posterior distributions given the observed values. Next the analysis model of interest is fitted in each imputed dataset and the multiple estimates are pooled using Rubin’s rules to obtain a final estimate [10].

Advantages of MI include an increased sample size compared with CCA, providing gains in precision, as well as the possibility to incorporate information from additional, so-called auxiliary variables, which may help to further increasing precision and reducing bias. The general assumptions under which MI is guaranteed to be theoretically unbiased are more relaxed than for CCA. One commonly used approach to implement MI with multiple incomplete variables is “fully conditional specification” (FCS) [12], where incomplete variables are imputed iteratively from univariate imputation models conditional on other variables in the imputation model. This is also known as “multiple imputation by chained equations” (MICE). One feature of FCS is that each univariate model may be flexibly specified to incorporate appropriate assumptions regarding each variable’s distribution and its relationship with other variables.

In the context of RCTs, it is recommended that MI be conducted separately by treatment group to reduce bias in ACE estimates [13]. In observational studies, the task of estimating causal effects may be conceptualised as emulating the design and analysis of the target trial. This raises the question of whether MI should be conducted by exposure group when estimating the ACE in observational studies. We designed a simulation study considering a range of scenarios to compare the performance of CCA and several MI methods when estimating the ACE using outcome regression. We considered MI on the whole dataset with or without exposure interaction terms in the imputation model, and MI stratified by exposure group. The simulations were based on an illustrative case study, which investigated the association between cannabis use in adolescence and depression and anxiety in young adulthood in females using data from the Victorian Adolescent Health Cohort Study (VAHCS) [14].

## Methods

### Motivating case study

The VAHCS is a cohort of 1,943 participants (1,000 females) recruited in Victoria when they were 14-15 years old, between 1992 and 1993. The study was approved by the Human Research Ethics Committee of the Royal Children’s Hospital. A survey was conducted every six months (waves two-six) over the three years after recruitment, and again when participants were 20-21 years old (young adulthood phase, wave seven). The frequency of cannabis use in the previous six months was self-reported at each wave. The exposure of interest was cannabis use in adolescence, with individuals defined as exposed if they used cannabis more than once a week in any wave of the adolescent period (waves two-six) and unexposed otherwise. The proportion exposed was 8.8% amongst the *n*=953 female participants who had complete data on the exposure and three of the confounders with very few missing values (specifically, 13 missing values in exposure, 34 missing values in parental education, 1 missing value in parental divorce and 6 missing values in antisocial behaviour — defined below). This henceforth defines the analytical sample for the case study.

The outcome was a measure of depression and anxiety at age 20-21 years (wave seven), assessed using the Computerised Revised Clinical Interview schedule (CIS-R) [15]. The case study used the log-transformed, standardised CIS-R total score as the outcome measure. A key confounder was adolescent depression and anxiety, defined as present if the CIS-R score was 12 or higher in any wave during the adolescent period, and absent otherwise [16]. Other confounders included: antisocial behaviour in any of the adolescent waves, assessed through a self-reported early delinquency scale that included property damage, interpersonal violence and theft [17]; alcohol use in any of the adolescent waves, based on self-reported frequency, with frequent drinkers defined as participants who drank three days or more in the previous week; parental divorce or separation by wave six; and parental education defined by high school education completion by wave six. Participants’ age at wave two, log-transformed and standardised, was used as an auxiliary variable for MI. Table 1 shows descriptive statistics and missing data proportions for the relevant variables in the analytical sample, as well as the notation used henceforth to refer to each variable. The proportion with complete records was 72.8%.

**Table 1:** Descriptive statistics for the analysis variables in the analytical sample for the case study, using the data from Victorian Adolescent Health Cohort Study (*n*=953)

Role	Label	Variable	Unexposed	Exposed	Missing (%)
			*n* (%) or mean (SD)^a^		
Exposure	*X*	Cannabis use, Yes	869 (91.2)	84 (8.8)	0
Outcome	*Y*	Adulthood mental health score^b^	-0.02 (0.97)	0.17 (1.30)	10.3
Confounder	$C_1$	Parents not completed high-school, Yes	322 (37.1)	35 (41.7)	0
Confounder	$C_2$	Parental divorce, Yes	169 (19.4)	38 (45.2)	0
Confounder	$C_3$	Antisocial behaviour, Yes	69 (7.9)	31 (36.9)	0
Confounder	$C_4$	Alcohol use, Yes	218 (31.1)	65 (87.8)	18.6
Confounder	$C_5$	Adolescent depression & anxiety, Yes	432 (56.5)	62 (81.6)	11.9
Auxiliary	*A*	Participant’s age at wave two^b^	-0.01 (1.00)	0.15 (0.95)	8.4

^a^For incomplete variables, the descriptive statistics are obtained from the records with available data on the given variable

^b^In standard deviation units, standardised to the combined sample

The estimand of interest was the ACE of cannabis use (*X*) in female adolescents on young adulthood mental health (*Y*), defined as the difference in the expected value of the potential outcomes under exposure versus under no exposure, denoted as $ACE=E[Y^{x=1}]-E[Y^{x=0}]$. Although debatable, for the purpose of our investigation of missing data methods, we assumed that, in the absence of missing data, the key causal assumptions of exchangeability given the vector of confounders $\boldsymbol{C}=(C_1,\dots ,C_5)$ (see Table 1), consistency, and positivity would hold in this case study. Additionally, we assumed no effect modification, i.e. constant effect within confounder strata [18, 19]. Under these assumptions, if there were no missing data, the ACE would be identified as:

$$\begin{aligned} ACE=E[Y\vert \boldsymbol{C}=\boldsymbol{c},X=1]-E[Y\vert \boldsymbol{C}=\boldsymbol{c},X=0], \end{aligned}$$

for any $\mathbf {c}$ such that $\Pr{}(\boldsymbol{C}=\boldsymbol{c})>0$, which could be estimated by positing a linear regression model for the outcome with no exposure-confounder interactions. We considered a linear regression with mean specified as:

1 $$\begin{aligned} E[Y|X,\boldsymbol{C}]=\theta _{0}+\theta _{1}X+\theta _{2} C_{1}+\theta _{3} C_{2}+\theta _{4} C_{3}+\theta _{5} C_{4}+\theta _{6} C_{5}. \end{aligned}$$

The estimate of the ACE is given by the estimated regression coefficient for the exposure, $\hat{\theta }_1$.

### Simulation study

In the simulation study, data generation was based on the VAHCS case study. Unless stated otherwise, all models used parameter values estimated from VAHCS. The simulation study assessed three exposure-prevalence scenarios with a progressive increase in the proportion exposed: 10%, 30% and 50% (it was 8.8% in VAHCS).

For the primary aim of evaluating the bias of causal effect estimates when conducting MI by exposure group relative to other implementations of MI when the exposure groups are relatively large, we considered sample sizes such that the smallest exposure group was larger than 100 observations across exposure-prevalence scenarios. The sample size in each case was chosen to produce approximately the same standard error for the ACE in each exposure-prevalence scenario, enabling comparison of the impact of bias on coverage probability across scenarios. Datasets contained 1,300 observations for the scenario with 10% exposed, 700 observations for the scenario with 30% exposed, and 550 observations for the scenario with 50% exposed. These specifications ensured that around 80% power was achieved when analysing the complete datasets in all scenarios.

There is a bias-variance trade-off in the use of MI by exposure group that is driven by the absolute size of the smallest exposure group. Therefore, to investigate the optimal size of the smallest exposure group for using this approach in terms of reduced bias and increased precision, a secondary set of simulations was performed, considering a range of sample sizes such that the datasets contained 500, 1,300, 2,000 and 2,750 observations with the exposure prevalence fixed at 10% (power ranging from 11.5% to 94.9%, see the Supplementary Material).

#### Complete data generation

The data generation procedure was initiated by separately generating the auxiliary variable (*A*) from the standard normal distribution and $C_1$ from a binomial distribution. Then all the other confounders were generated following the order listed in Table 1, followed by the exposure, each conditional on the previously generated variables. All were binary and were generated from a binomial distribution with probability defined by a logistic regression model.

Seven scenarios were considered for generating the outcome *Y*. Specifically, values were drawn from a linear regression model with mean specified as per Eq. (2), with $\delta$ reflecting the strength of the interaction between the exposure *X* and the (strong) confounder $C_5$:

2 $$\begin{aligned} E[Y|X,\boldsymbol{C}]=\alpha _{0}+\alpha _{1}C_{1}+\alpha _{2}C_{2}+\alpha _{3}C_{3}+\alpha _{4}C_{4}+\alpha _{5}C_{5}+\alpha _{6}X+\delta \alpha _{6}X\times C_{5}. \end{aligned}$$

The values of $\delta$ were set to 0, $\pm 0.25$, $\pm 0.5$, and $\pm 0.75$ for no, weak, moderate, and strong positive/negative interaction.

The target analysis was the outcome regression model without interactions in (1) and the target estimand was the exposure coefficient $\theta _1$ in (1), which is equal to the ACE under the assumption of no effect modification. The target analysis model was mis-specified in the scenarios where the outcome generation model (2) had interactions.

To make the results comparable across the interaction scenarios in the outcome model, we set the target value of the estimand ($\theta _1$) to 0.3 in each case by tweaking the main effect $\alpha _6$ in (2).

#### Missing data generation

There were four incomplete variables in the case study: the auxiliary variable, *A*, two confounders, $C_4$ and $C_5$, and the outcome, *Y*. In the simulation study, we did not generate missing data in the auxiliary variable *A*. We considered two scenarios for missing data: one with incomplete outcome only and the other with incomplete outcome and confounders ($C_4$ and $C_5$). For each incomplete variable, missingness was imposed by drawing the value of a missingness indicator, which equals one if the variable is missing and zero otherwise.

For the scenario where only the outcome had missing values, the missingness indicator $M_{Y}$ was generated using the following logistic model:

$$\begin{aligned} \text {logit}\Pr {}(M_{Y}=1)=\beta _{0}+0.030A+\log(3)X+\beta _{1}C_{5}+\beta _{2}X\times C_{5}. \end{aligned}$$

We considered three missingness scenarios: (i) missingness depended only on the exposure, i.e. $\beta _1=\beta _2=0$; (ii) missingness depended on the exposure and the strong confounder $C_5$, i.e. $\beta _1=\log (3), \beta _2=0$; and (iii) missingness depended on the exposure, $C_5$, and the exposure interaction with $C_5$, i.e. $\beta _1=\log (3), \beta _2=\log (2)$. The missingness proportion in each scenario was controlled to be 30% by searching over a grid of $\beta _0$ values.

For the scenario where the outcome and the two confounders had missing values, missingness indicators for $C_4$, $C_5$ and *Y* were generated sequentially. The missingness indicator for $C_4$ was generated from a model that depended on *A* and *X*:

$$\begin{aligned} \text {logit}\Pr {}(M_{C_4}=1)=\gamma _{0}+0.323A-0.624X. \end{aligned}$$

The model for generating $M_{C_5}$ included *A* as well as $M_{C_4}$ to control the overlap between missingness in $C_4$ and $C_5$ to be as it was observed in VAHCS:

$$\begin{aligned} \text {logit}\Pr {}(M_{C_{5}}=1)=\zeta _0-0.029A+3.835M_{C_{4}}+\log(3)X+\zeta _{1}C_{5}+\zeta _{2}X\times C_{5}. \end{aligned}$$

Similarly, the model for generating the missingness indicator for *Y* included $M_{C_4}$ and $M_{C_5}$:

$$\begin{aligned} \text {logit}\Pr {}(M_{Y}=1)&=\eta _{0}-0.025A+0.685M_{C_4}+0.658M_{C_5}\\&+\log(3)X+\eta _{1}C_{5}+\eta _{2}X\times C_{5}. \end{aligned}$$

Similar to the scenario where only the outcome had missing values, three scenarios were considered for generating $M_{C_5}$ and $M_{Y}$: (i) missingness depended only on the exposure, i.e. $\zeta _1=\eta _1=\zeta _2=\eta _2=0$; (ii) missingness depended on the exposure and the strong confounder $C_5$, i.e. $\zeta _1=\eta _1=\log (3),\zeta _2=\eta _2=0$; (iii) missingness depended on the exposure, $C_5$, and their interaction, i.e. $\zeta _1=\eta _1=\log (3),\zeta _2=\eta _2=\log (2)$.

By searching over a grid of values for $\beta _0$, $\zeta _0$ and $\eta _0$, the missingness proportions for the incomplete variables were controlled to be 10% in $C_4$, 10% in $C_5$ and 20% in *Y*. The proportion of records with any missing data was 30% in all simulation scenarios (in the case study it was 27.2%).

#### Missing data methods

The FCS approach models the distribution of each incomplete variable conditional on other variables in the imputation model and possible interactions [12]. The imputation procedure creates a number of imputed datasets, each obtained by iterating over the conditional imputation models. Specifically, the iterative process is started by randomly filling all missing values, then sequentially fitting the imputation models on each incomplete variable using the observed records and current imputations of other variables, and drawing imputed values. The fitting-drawing cycle is repeated on all incomplete variables until convergence to obtain one imputed dataset. The analysis model is then applied to each imputed dataset, and the results are combined using Rubin’s rules: the multiple parameter estimates obtained are combined into a final estimate by taking the average and a variance estimator is obtained that incorporates both within-imputation (sampling) variance and between-imputation (missing data uncertainty) variance [10].

The considered methods for handling missing data were CCA, MI conducted separately within each exposure group (MI-EG), and a series of MI approaches that were conducted on the whole sample and differed in the interaction terms within the univariate models of FCS: no interactions (MI-NI); an exposure-outcome interaction (MI-E$\times$O); an exposure-confounder $C_5$ interaction (MI-E$\times$C); both exposure-outcome and exposure-confounder $C_5$ interactions (MI-E$\times$OC); and interactions between exposure and all incomplete variables (MI-E$\times$I). For the scenario with only the outcome incomplete, MI-E$\times$O was equivalent to MI-NI, and MI-E$\times$OC and MI-E$\times$I were both equivalent to MI-E$\times$C, therefore the number of considered methods was reduced.

All MI approaches were carried out using the ‘mice’ package in R with five iterations [20]. The imputation method used linear regression for the continuous variable (outcome), and logistic regression for the binary variables (the confounders). Imputation predictors included all analysis variables and the auxiliary variable *A*, as well as the interaction terms outlined for each approach. Following a common rule of thumb [21], the number of imputations was 30 for all MI approaches in the whole cohort to reflect the proportion of missing data, and larger for the MI-EG approach since there was a higher missingness proportion in the exposed sample, see the Supplementary Material.

#### Performance indicators

We generated and analysed 2,000 datasets for each scenario. We reported for each method the mean of the $\theta _1$ estimates and corresponding Monte Carlo standard errors (MCSE); the bias, defined as the difference between the mean of the $\theta _1$ estimates and the target value of $\theta _1$ (0.3), in both absolute and relative terms (i.e. as a percentage); the mean squared error (MSE), given by the sum of the squared bias and variance of the 2,000 estimates; the empirical standard error (EmpSE), given by the square root of the variance of the 2,000 estimates, and its MCSE; the model-based standard error (ModSE), given by the average of 2,000 estimated standard errors; and the coverage probability, estimated by the proportion of the 95% confidence intervals that contained the target estimand ($\theta _1$) across the 2,000 datasets, and its MCSE.

### Case study analysis

We also applied the described missing data methods to the case study. MI used logistic regression for incomplete binary variables ($C_4$ and $C_5$), and linear regression for the incomplete continuous variables (*A* and *Y*). One hundred imputations were performed, each based on ten ‘mice’ iterations.

All analyses were conducted in R version [3.6.1].

## Results

### Simulation study results

**Fig. 1:**
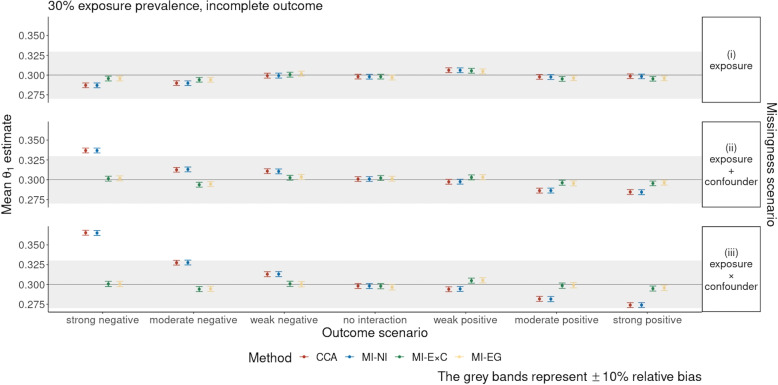
Simulation study results: mean of the $\theta _1$ estimates and Monte Carlo standard errors for the scenario with 30% exposure prevalence and an incomplete outcome only

**Fig. 2:**
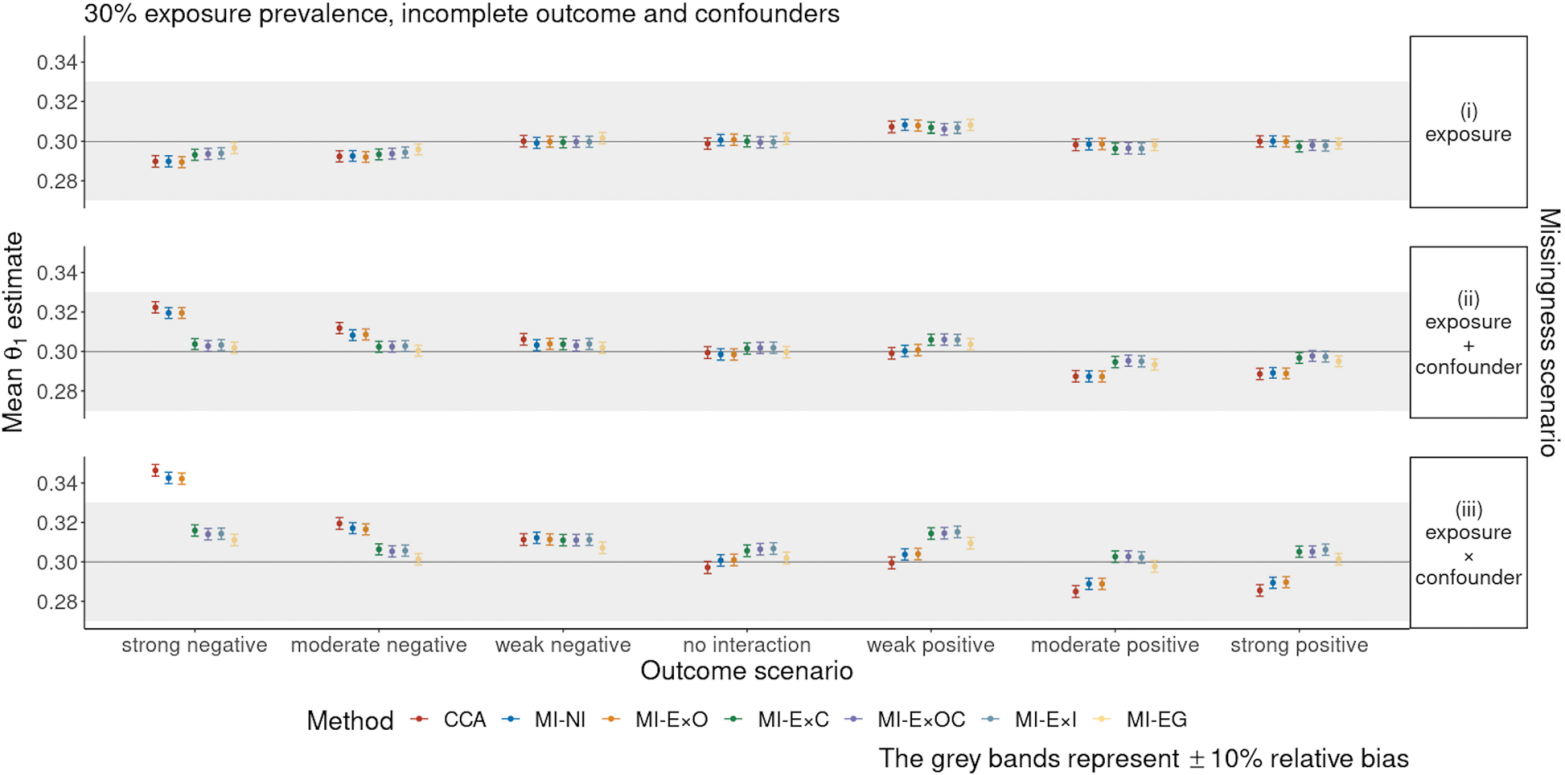
Simulation study results: mean of the $\theta _1$ estimates and Monte Carlo standard errors for the scenario with 30% exposure prevalence and incomplete outcome and confounders

**Fig. 3:**
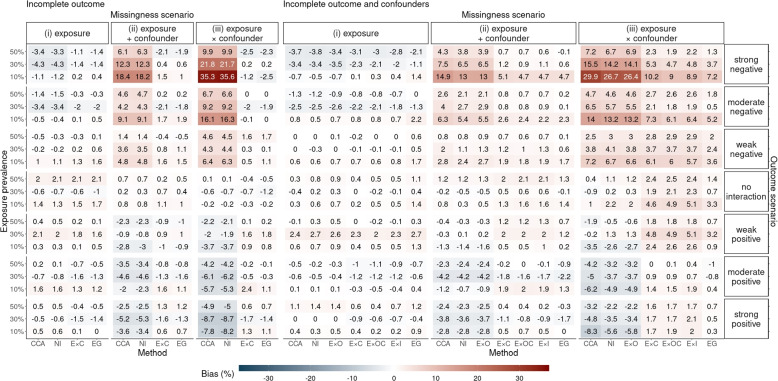
Simulation study results: relative bias of the complete case analysis (CCA) and six multiple imputation (MI) methods in estimating $\theta _1$ across all missingness, outcome and exposure-prevalence scenarios

**Fig. 4:**
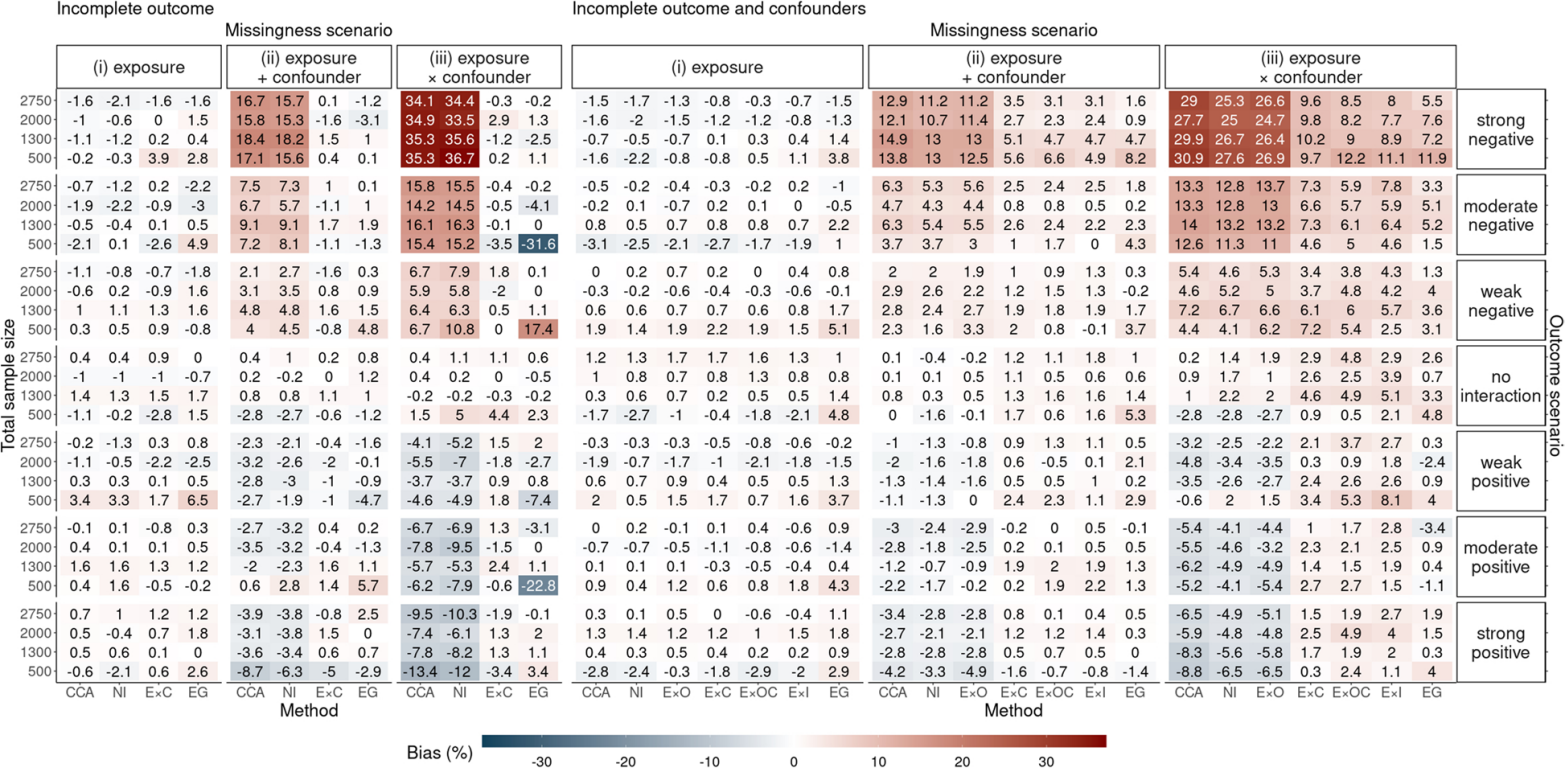
Secondary set of simulation study results: relative bias of the complete case analysis (CCA) and six multiple imputation (MI) methods in estimating $\theta _1$ for different sample sizes across missingness and outcome scenarios with 10% exposure prevalence

**Fig. 5:**
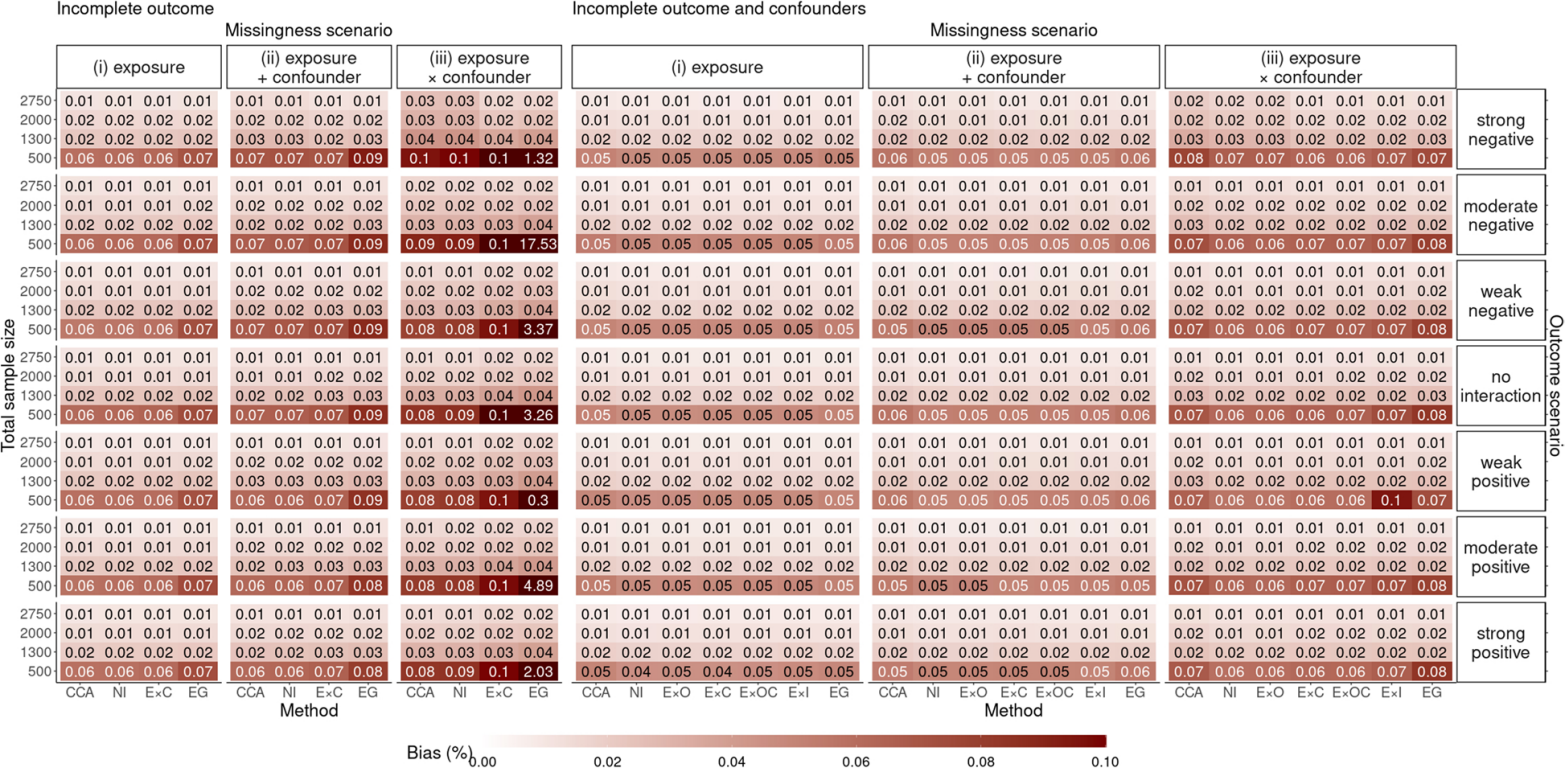
Secondary set of simulation study results: Mean squared error (MSE) of the complete case analysis (CCA) and six multiple imputation (MI) methods in estimating $\theta _1$ for different sample sizes across missingness and outcome scenarios with 10% exposure prevalence

In the primary set of simulations, the patterns of the results under the three exposure-prevalence scenarios were similar but differed in the extent of biases. To simplify exposition, we focus on the results from the 30% exposure prevalence scenario in Figs. 1 and 2, providing details for the other scenarios in Figs. 3, 4 and 5 and the Supplementary Material. Figure 1 shows the mean of the $\theta _1$ estimates across the 2,000 simulated datasets, and corresponding MCSEs, for the four missing data methods when only the outcome was incomplete.

The estimates given by all methods were approximately unbiased in missingness scenario (i) (missingness only depended on the exposure, maximum ± 3.3% relative bias). In missingness scenarios (ii) (missingness depended on the exposure and strong confounder) and (iii) (missingness depended on the exposure, strong confounder, and their interaction), CCA and MI-NI overestimated $\theta _1$ in scenarios where the outcome model included a negative interaction between the exposure and strong confounder, and underestimated $\theta _1$ in the positive interaction scenarios. The relative bias given by MI-NI in missingness scenario (iii) was up to 35.6% in the strong negative interaction outcome scenario and −8.2% in the strong positive interaction outcome scenario. In contrast, MI-E$\times$C and MI-EG were mostly unbiased (less than 3.8% relative bias across seven outcome scenarios) in missingness scenarios (ii) and (iii).

Figure 2 shows the performance for all seven methods for handling missing data for the scenario with incomplete outcome and confounders. Here too, substantial biases were observed in the CCA and MI-NI approaches. Methods MI-E$\times$C, MI-E$\times$OC, MI-E$\times$I and MI-EG showed generally similar patterns across the seven outcome scenarios: estimates were slightly biased (relative bias ranging from −2.0% to 5.9%). The common feature of these methods is that they include (explicitly or implicitly) an interaction between the exposure and the confounder $C_5$ in the univariate imputation models. In contrast, MI-E$\times$O did not perform as well as the other MI methods incorporating interactions and had similar performance to MI-NI, with the relative bias being −3.2% in the strong negative interaction outcome scenario and 13.5% in the strong positive interaction outcome scenario, in missingness scenario (iii).

Figure 3 show the relative bias across all scenarios (including scenarios where the exposure prevalence was 10% or 50%), which shows bias was inversely related to the exposure prevalence: the higher the prevalence, the lower the bias.

The model-based standard errors were close to the empirical standard errors across all scenarios (difference less than 0.01). For this reason, the coverage of the confidence intervals among the less biased methods was approximately at the nominal 95% level (see the Supplementary Material). The MSEs were close to zero across the methods (ranging from 0.014 to 0.043). MI-EG yielded similar MSEs compared with other methods for 30% and 50% exposure prevalence scenarios.

In the secondary set of simulations, we investigated the optimal size of the smallest exposure group in 10% exposure prevalence for conducting MI-EG. Figures 4 and 5 show the relative bias and MSE results, respectively, across missing data approaches and sample sizes. For moderate sample sizes of 1,300 observations or larger, both MI-EG and MI-EC were approximately unbiased without substantial increase in the MSE. However, when the total sample size was 500 (the size of exposed group was around 50), the results indicated that MI-EG was biased and yielded the largest MSE among all approaches. Meanwhile MI-EC was approximately unbiased but still exhibited considerable MSE in this setting.

### Case study results

Table 2 shows the estimates of $\theta _1$ obtained by applying the seven missing data methods in the case study.

**Table 2:** Estimates of $\theta _1$ obtained using various missing data methods in the case study (*n*=953)

Method	$\theta _1$ estimate	Standard error	*P*-value	95% confidence interval
CCA	0.282	0.136	0.038	0.016, 0.549
MI-NI	0.296	0.124	0.018	0.051, 0.540
MI-E$\times$O	0.301	0.123	0.015	0.059, 0.544
MI-E$\times$C	0.301	0.127	0.018	0.051, 0.551
MI-E$\times$OC	0.301	0.122	0.014	0.062, 0.540
MI-E$\times$I	0.294	0.121	0.015	0.057, 0.532
MI-EG	0.273	0.122	0.026	0.033, 0.514

The estimates given by all seven methods suggest a moderate negative effect of cannabis use in adolescence on young adulthood mental health in females. The estimates across all methods were similar, although the precision of CCA was slightly lower.

## Discussion

This work examined methods for handling missing data when estimating causal effects using outcome regression without interactions, an approach that is widely used in epidemiological research. By conducting simulations in a wide range of scenarios, our study showed that when the exposure groups are relatively large, implementing MI separately by exposure group (MI-EG) was the approach that led to the least bias across all scenarios, and was approximately unbiased in most scenarios, with MI including the exposure-confounder interaction being the next best approach.

### Comparison of results with previous literature

The finding that MI-EG had the best performance is consistent with previous research in the randomised trial context. Sullivan et al. [13] investigated the performance of MI conducted separately by treatment group when using outcome regression to estimate the average treatment effect in an RCT, when missingness in the outcome depended on baseline covariates only, on both baseline covariates and treatment (equivalent to our missingness scenario (ii)), or on both of these and their interaction (missingness scenario (iii)). Their results showed that MI by treatment group was unbiased, whereas CCA and MI with no interactions (MI-NI in our study) were biased.

In the context of an analysis model with exposure-confounder interactions, Tilling et al. [22] assessed MI within subsamples defined by either exposure or confounder groups, and several MI approaches incorporating interactions in the imputation models. Their results showed that ignoring interactions in MI can lead to biased estimates or over-coverage of the confidence intervals. Our study extends these results, showing that allowing for interactions in the imputation step is important even if the analysis model does not include an interaction. In the following paragraphs we provide further discussion and comparison of our results with previous theoretical work.

Several authors have discussed the bias of MI inferences when the imputation and analysis model are not compatible, meaning that they cannot be derived from an overarching joint model [23, 24]. Many simulation studies [22, 24, 25] have shown that estimates can be unbiased under incompatibility if the analysis model is correctly specified and the imputation model can be made compatible with the analysis model by setting one or more parameters to zero, as discussed by Bartlett et al [24]. This aligns with our findings for MI approaches with interactions in the no-interaction outcome generation model scenario, in which the analysis model is correctly specified and the imputation model can be made compatible by setting the coefficients of interaction terms to zero.

These previous studies did not discuss compatibility if the analysis model is misspecified, as was the case in our outcome scenarios with interactions, but the imputation model (or its restricted version, e.g. MI-E$\times$C is the restricted version of MI-E$\times$OC and MI-E$\times$I) correctly specifies the interactions. In our study, in the outcome scenarios with interactions, we found that the correctly specified imputation model (MI-E$\times$C) and its less restrictive versions (MI-E$\times$OC and MI-E$\times$I) led to unbiased estimates even if these were incompatible with the misspecified analysis model. In contrast, the approach where the imputation model (along with the analysis model) was misspecified in the sense of the interaction specification failed to yield unbiased estimates (MI-NI and MI-E$\times$O).

In a setting with an incomplete outcome only, and where the missingness does not depend on the outcome itself, CCA has been shown to be unbiased if the regression analysis model is correctly specified and includes the missingness predictors as adjustment variables [26–28]. Within this scenario, the regression coefficient and variance estimators given by our MI-NI approach have been proven to be asymptotically equivalent to CCA [7, 13, 29]. We found that both CCA and MI-NI also performed similarly and were approximately unbiased when the analysis model was misspecified (i.e. in the scenarios where the outcome generation model included an exposure-confounder interaction), and also when confounders were incomplete, as long as the missingness did not depend on the confounders or outcome given the exposure and an auxiliary variable. Indeed, we found that CCA and MI-NI were approximately unbiased in missingness scenario (i) where this holds.

In the scenario with incomplete outcome and confounders and where there was no exposure-confounder interaction in the outcome generation model, all methods were approximately unbiased, regardless of the missingness scenario. These results align with findings from Moreno-Betancur et al. [30] who, using missingness directed acyclic graphs (m-DAGs), proved that the conditional distribution of the outcome can be estimated unbiasedly by fitting a correctly specified regression model to the complete cases if missingness in exposure, outcome and confounders are not caused by the outcome, corresponding to their m-DAG E. An m-DAG for our missingness scenario (ii) can be obtained from m-DAG E in Moreno-Betancur et al. [30] by removing arrows towards the exposure missingness indicator. Thus, by Lemma 4 given by Mohan [31], the conditional distribution of the outcome is also recoverable in our missingness scenario (ii) and can be estimated unbiasedly similarly, by fitting a correctly specified regression model to the complete cases. Interestingly, similar results were found in missingness scenario (iii).

### Strengths and limitations

Our simulation study involved the generation of data closely resembling a real case study and assessed a wide range of scenarios (a total of 252). Still, our study does not cover all possible scenarios, and further investigations considering other scenarios would be worthwhile, specifically considering settings with a binary outcome or continuous confounders [22, 25]. Given findings from Sullivan et al. [13] in a trial setting, which were similar for both continuous and binary outcomes, we conjecture the bias reduction provided by MI-EG and MI-E$\times$G would also apply to binary outcomes analysed using logistic or log-binomial regression. However, in the case of logistic regression, we do not expect the same pattern of bias in biased methods (CCA, MI-NI and MI-E$\times$O) across scenarios going from strong negative to strong positive interactions in the outcome model, because of the non-collapsibility of the odds ratio [32, 33]. Additionally, further research investigating incomplete exposure settings might assist in guiding practice, especially regarding how to impute the exposure. Moreover, the confounder used as the missingness predictor was the same as that which interacted with the exposure in the outcome generation. Further research on method performance when these key covariates are distinct is warranted. Our study focused on outcome regression as a confounding adjustment method, which assumes a constant effect size across confounder strata. However, there are other confounding adjustment methods that do not rely on such assumptions, such as inverse probability weighting (IPW) and g-computation. These methods provide estimates of the marginal average causal effect across confounder strata, which in particular helps circumvent issues arising due to non-collapsibility when using the odds ratio as effect measure in the case of binary outcomes [32, 33]. Although research on methods for handling missing data with IPW is available [34–36], consideration of MI-EG still requires investigation, and research assessing MI-based approaches for g-computation is ongoing [37]. Finally, it would be useful to investigate the performance of MI-EG in the context of other MI approaches, such as multivariate normal imputation [38] and substantive-model-compatible fully conditional specification [24].

### Implications for practice

Despite MI-EG yielding the least biased estimates, this approach may have limited applicability. First, the exposure must be complete. Second, MI within subsamples defined by exposure status may encounter numerical issues particularly for low-prevalence exposures. Random violations of positivity are more likely to happen with lower exposure prevalence and multi-category exposures. Additionally, our recommendation is to avoid using the MI-EG approach when the smallest exposure group is relatively small (severe bias and loss in precision seen when the smallest group had 50 observations in our secondary set of simulations, Figs. 4 and 5). Third, it is unclear how the approach should be applied in the continuous exposure setting. Any categorisation of the continuous exposure to split the dataset would result in categories that still retain heterogeneity in the exposure, so the necessary interactions may not be fully accounted for [22, 25]. For the above cases, MI with exposure-confounder interactions, which also performed well in our simulations, could be considered as an alternative approach.

Overall, our recommendation for practice is to conduct MI by exposure group when MI is used to handle missing data in the context of causal inference using outcome regression when the exposure groups are relatively large. If conducting MI by exposure group is not feasible, then we recommend including exposure-confounder interactions in the imputation models as the next-best option.

## Supplementary Information

**Additional file 1.**

## Data Availability

The data underlying this article will be shared on reasonable request to the corresponding author with the permission of the study custodian. The data and code for simulation study are available here https://github.com/Jiaxin-Zhang-GitHub/Should-multiple-imputation-be-stratified-by-exposure-group-when-estimating-causal-effects-via-outcom.git.

## Abbreviations

CCA: Complete-case analysis
MI: Multiple imputation
RCT: Randomized controlled trials
ACE: Average causal effect
FCS: Fully conditional specification
MICE: Multiple imputation by chained equations
VAHCS: Victorian Adolescent Health Cohort Study
CIS-R: Computerized Revised Clinical Interview Schedule
SD: Standard deviation
MI-EG: Multiple imputation conducted separately within each exposure group
MI-NI: Multiple imputation with no interactions
MI-E$\times$O: Multiple imputation with exposure-outcome interaction
MI-E$\times$C: Multiple imputation with exposure-confounder C_5_ interaction
MI-E$\times$OC: Multiple imputation with both exposure-outcome interaction and exposure-confounder C_5_ interaction
MI-E$\times$I: Multiple imputation with interactions between exposure and all incomplete variables
MCSE: Monte Carlo standard error
EmpSE: Empirical standard error
ModSE: Model-based standard error
m-DAG: Missingness directed acyclic graphs
