# Supplementary material for: Should multiple imputation be stratified by exposure group when estimating causal effects via outcome regression in observational studies?
Source: BMC Med Res Methodol. 2023 Feb 16;23:42. doi: 10.1186/s12874-023-01843-6 (PMC9933305; doi:10.1186/s12874-023-01843-6)
Supplement: Supplementary file 1 — Additional file 1. [file 12874_2023_1843_MOESM1_ESM.pdf]

Supplementary information for
"Should multiple imputation be stratified by exposure group when estimating causal effects via outcome regression in observational studies?", BMC Medical Research Methodology

Jiaxin Zhang[1,2,*], S. Ghazaleh Dashti[2,1], John B. Carlin[2,1], Katherine J. Lee[2,1], Margarita Moreno-Betancur[1,2]
[1] Clinical Epidemiology and Biosatistics Unit, Department of Paediatrics, University of Melbourne, Australia
[2] Clinical Epidemiology and Biosatistics Unit, Murdoch Children's Research Institute, Australia
[*] jiaxizhang1@unimelb.edu.au

The contents of this document are as follows:

- Table S1 Parameter values used for generating complete datasets in the simulation study.
- Table S2 Regression coefficient values used in logistic model for generating missingness indicators in the simulation study.
- Table S3 Number of imputations for MI-EG approach.
- Table S4-9 Performance of the complete case analysis (CCA) and six multiple imputation (MI) methods in estimating θ_1 under missingness scenario A, B and C.
- Figure S1 The mean squared error across all missingness, outcome and exposure-prevalence scenarios.
- Figure S2 Power estimates across the missing data methods, for four total sample sizes in 10% exposure prevalence.

Table S1: Parameter values used for generating complete datasets in the simulation study

Variable generated	Distribution or regression coefficient values								
A	$N(0, 1)$								
C_1	$B(n, 0.375)$[a]								
	intercept	A	C_1	C_2	C_3	C_4	C_5	X	$X \times C_5$
C_2	-1.448	0.051	0.433	-	-	-	-	-	-
C_3	-2.279	0.151	-0.264	0.74	-	-	-	-	-
C_4	0.067	-0.018	-0.113	0.846	1.118	-	-	-	-
C_5	-1.602	-0.061	0.271	0.631	0.939	0.983	-	-	-
10% exposure prevalence scenario									
X	-4.717	0.228	-0.061	1.168	1.650	0.420	2.492	-	-
Y									
Interaction scenario:									
Strong negative	-0.512	-	0.072	0.129	0.066	0.723	0.031	0.744	-0.558
Moderate negative	-0.512							0.498	-0.249
Weak negative	-0.512							0.375	-0.094
No interaction	-0.508							0.300	-
Weak positive	-0.512							0.250	0.063
Moderate positive	-0.512							0.215	0.107
Strong positive	-0.512							0.188	0.141
30% exposure prevalence scenario									
X	-2.699	0.228	-0.061	1.168	1.650	0.420	2.492	-	-
Y									
Interaction scenario:									
Strong negative	-0.512	-	0.072	0.129	0.066	0.723	0.031	0.599	-0.499
Moderate negative	-0.512							0.449	-0.225
Weak negative	-0.512							0.361	-0.090
No interaction	-0.508							0.299	-
Weak positive	-0.512							0.262	0.066
Moderate positive	-0.512							0.224	0.112
Strong positive	-0.512							0.201	0.151

50% exposure prevalence scenario									
X	-1.346	0.228	-0.061	1.168	1.650	0.420	2.492	-	-
Y									
Interaction scenario:									
Strong negative	-0.512							0.510	-0.382
Moderate negative	-0.512							0.413	-0.207
Weak negative	-0.512							0.348	-0.087
No interaction	-0.508	-	0.072	0.129	0.066	0.723	0.031	0.300	-
Weak positive	-0.512							0.264	0.066
Moderate positive	-0.512							0.235	0.118
Strong positive	-0.512							0.213	0.160

[a]The binary confounder C_1 was generated from the Binomial distribution with n=1300, 700, 550 for the scenarios with 10%, 30% and 50% exposed, respectively.

Table S2: Regression coefficient values used in logistic model for generating missingness indicators in the simulation study

	intercept			A	M_{C_4}	M_{C_5}	X	C_5	$X \times C_5$	Missingness
	X=10%	X=30%	X=50%							
incomplete outcome										
	-0.973	-1.222	-1.458				log(3)	-	-	
M_Y	-1.670	-1.932	-2.174	0.030	-	-	log(3)	log(3)	-	30%
	-1.733	-2.141	-2.514				log(3)	log(3)	log(2)	
incomplete confounders and outcome										
M_{C_4}	-2.185	-2.076	-1.957	0.323	-	-	-0.624	-	-	10%
	-3.340	-3.568	-3.778				log(3)	-	-	
M_{C_5}	-4.045	-4.286	-4.494	-0.029	3.835	-	log(3)	log(3)	-	10%
	-4.178	-4.574	-4.870				log(3)	log(3)	log(2)	
	-1.696	-1.957	-2.189				log(3)	-	-	
M_Y	-2.429	-2.708	-2.943	-0.025	0.685	0.658	log(3)	log(3)	-	20%
	-2.528	-2.993	-3.357				log(3)	log(3)	log(2)	

Table S3: Number of imputations for MI-EG approach

Exposure prevalence	10%	30%	50%
Number of imputations	65	50	40

Table S4: Performance of the complete case analysis (CCA) and six multiple imputation (MI) methods in estimating θ_1 under missingness scenario (i-iii) with 10% exposure prevalence and incomplete outcome.

Outcome scenario	Method	Missingness scenario[a]								
		(i): exposure			(ii): exposure + confounder			(iii): exposure × confounder		
		Bias (%)[b]	EmpSE[c]	Coverage[d]	Bias (%)[b]	EmpSE[c]	Coverage[d]	Bias (%)[b]	EmpSE[c]	Coverage[d]
Strong negative	CCA	-1.101	0.147	94.9	18.356	0.156	93.2	35.293	0.177	90.2
	MI-NI[e]	-1.216	0.149	94.9	18.183	0.157	93.0	35.600	0.178	90.6
	MI-E×C[e]	0.246	0.149	95.5	1.462	0.157	94.7	-1.221	0.188	95.4
	MI-EG[e]	0.404	0.152	94.9	0.975	0.160	95.3	-2.480	0.198	95.8
Moderate negative	CCA	-0.528	0.143	95.6	9.115	0.153	94.8	16.129	0.167	94.5
	MI-NI	-0.357	0.144	95.3	9.054	0.154	95.0	16.324	0.169	94.8
	MI-E×C	0.140	0.146	95.0	1.654	0.157	95.2	-0.127	0.183	94.9
	MI-EG	0.469	0.148	95.8	1.872	0.162	95.3	-0.041	0.194	96.0
Weak negative	CCA	1.015	0.149	94.5	4.771	0.155	94.5	6.446	0.168	95.5
	MI-NI	1.063	0.151	94.7	4.756	0.157	94.5	6.329	0.171	95.8
	MI-E×C	1.317	0.151	94.8	1.576	0.160	95.3	0.543	0.182	95.8
	MI-EG	1.602	0.154	94.7	1.511	0.163	95.3	1.078	0.194	96.0
No interaction	CCA	1.406	0.151	93.4	0.799	0.156	95.1	-0.239	0.179	94.4
	MI-NI	1.329	0.153	93.3	0.764	0.158	94.2	-0.160	0.181	94.4
	MI-E×C	1.463	0.153	94.0	1.121	0.159	95.0	-0.317	0.195	94.5
	MI-EG	1.693	0.156	94.1	0.981	0.164	95.3	-0.167	0.205	93.4
Weak positive	CCA	0.279	0.147	94.9	-2.842	0.159	94.1	-3.707	0.173	95.1
	MI-NI	0.310	0.148	94.8	-2.968	0.160	93.9	-3.735	0.176	95.3
	MI-E×C	0.113	0.150	94.7	-0.961	0.162	94.5	0.928	0.187	95.4
	MI-EG	0.470	0.153	95.1	-0.870	0.168	94.7	0.774	0.200	94.9
Moderate positive	CCA	1.614	0.146	95.6	-2.012	0.157	94.1	-5.665	0.176	94.8
	MI-NI	1.611	0.147	95.6	-2.301	0.159	93.8	-5.322	0.177	94.9
	MI-E×C	1.270	0.148	95.7	1.648	0.160	94.0	2.434	0.191	95.1
	MI-EG	1.245	0.150	96.1	1.100	0.165	94.2	1.133	0.203	95.0
Strong positive	CCA	0.542	0.143	95.6	-3.577	0.155	95.2	-7.770	0.173	95.0
	MI-NI	0.606	0.144	96.0	-3.417	0.156	95.0	-8.167	0.175	95.2
	MI-E×C	0.122	0.144	96.0	0.605	0.159	95.4	1.301	0.187	95.5
	MI-EG	0.045	0.149	96.0	0.739	0.163	95.5	1.150	0.196	95.5

[a] Missingness scenario (i): missingness only depended on the exposure; missingness scenarios (ii): missingness depended on the exposure and strong confounder; and missingness scenario (iii): missingness depended on the exposure, strong confounder, and their interaction.
[b] Relative bias: the difference between the mean of the θ_1 estimates and the target value of θ_1(0.3), expressed as a percentage of the true value.
[c] Empirical standard error (EmpSE) from 2000 estimated regression coefficients, and Monte-Carlo standard error (MCSE) of EmpSE less than 0.005.
[d] Coverage probability, estimated by the proportion of the 95% confidence intervals that contained the target value of the ACE across the 2000 datasets, and MCSE of coverage less than 0.008.
[e] Abbreviations: NI: no interaction, E×C: exposure-confounder interaction, EG: by exposure group.

Table S5: Performance of the complete case analysis (CCA) and six multiple imputation (MI) methods in estimating θ_1 under missingness scenario (i-iii) with 30% exposure prevalence and incomplete outcome.

Outcome scenario	Method	Missingness scenario[a]								
		(i): exposure			(ii): exposure + confounder			(iii): exposure × confounder		
		Bias (%)[b]	EmpSE[c]	Coverage[d]	Bias (%)[b]	EmpSE[c]	Coverage[d]	Bias (%)[b]	EmpSE[c]	Coverage[d]
Strong negative	CCA	-4.298	0.134	94.8	12.294	0.137	94.5	21.803	0.140	92.1
	MI-NI[e]	-4.315	0.135	95.1	12.284	0.138	94.4	21.702	0.142	92.3
	MI-E×C[e]	-1.444	0.135	95.5	0.443	0.137	95.4	0.197	0.147	94.7
	MI-EG[e]	-1.438	0.136	94.7	0.556	0.139	95.2	0.162	0.150	95.2
Moderate negative	CCA	-3.371	0.131	95.5	4.162	0.134	95.4	9.160	0.137	95.2
	MI-NI	-3.413	0.132	95.7	4.323	0.135	95.4	9.226	0.137	95.4
	MI-E×C	-1.952	0.132	95.6	-2.128	0.136	95.2	-1.986	0.142	96.0
	MI-EG	-1.954	0.134	95.6	-1.842	0.139	95.7	-1.859	0.145	95.9
Weak negative	CCA	-0.227	0.139	94.1	3.575	0.135	94.1	4.306	0.139	95.1
	MI-NI	-0.199	0.139	94.1	3.479	0.137	94.2	4.352	0.140	95.1
	MI-E×C	0.201	0.140	94.0	0.828	0.137	95.2	0.256	0.145	95.5
	MI-EG	0.617	0.141	94.4	1.140	0.139	94.8	0.081	0.148	95.6
No interaction	CCA	-0.637	0.132	95.8	0.248	0.134	95.4	-0.644	0.143	94.7
	MI-NI	-0.680	0.133	95.6	0.271	0.135	95.3	-0.667	0.144	94.5
	MI-E×C	-0.648	0.133	96.1	0.712	0.136	95.4	-0.741	0.147	95.0
	MI-EG	-1.025	0.135	95.9	0.384	0.138	95.5	-1.193	0.153	94.5
Weak positive	CCA	2.058	0.134	94.8	-0.879	0.138	94.5	-2.037	0.139	95.3
	MI-NI	2.031	0.135	95.3	-0.825	0.139	93.8	-1.873	0.140	96.0
	MI-E×C	1.829	0.136	95.2	0.949	0.141	94.5	1.598	0.146	94.8
	MI-EG	1.560	0.137	95.3	1.032	0.143	94.6	1.759	0.148	95.3
	CCA	-0.743	0.134	95.2	-4.626	0.137	94.7	-6.100	0.145	94.1

Moderate positive	MI-NI	-0.798	0.135	95.4	-4.593	0.138	95.1	-6.187	0.146	93.9
	MI-E×C	-1.600	0.135	95.3	-1.291	0.141	95.1	-0.523	0.149	94.9
	MI-EG	-1.317	0.136	95.3	-1.567	0.142	95.3	-0.301	0.154	94.8
Strong positive	CCA	-0.465	0.131	95.2	-5.189	0.133	95.4	-8.715	0.137	95.3
	MI-NI	-0.562	0.132	95.1	-5.251	0.134	95.1	-8.684	0.137	95.5
	MI-E×C	-1.548	0.132	95.8	-1.580	0.136	95.9	-1.746	0.144	95.7
	MI-EG	-1.376	0.135	95.1	-1.258	0.137	95.8	-1.450	0.147	95.4

[a] Missingness scenario (i): missingness only depended on the exposure; missingness scenarios (ii): missingness depended on the exposure and strong confounder; and missingness scenario (iii): missingness depended on the exposure, strong confounder, and their interaction.
[b] Relative bias: the difference between the mean of the θ_1 estimates and the target value of θ_1(0.3), expressed as a percentage of the true value.
[c] Empirical standard error (EmpSE) from 2000 estimated regression coefficients, and Monte-Carlo standard error (MCSE) of EmpSE less than 0.004.
[d] Coverage probability, estimated by the proportion of the 95% confidence intervals that contained the target value of the ACE across the 2000 datasets, and MCSE of coverage less than 0.008.
[e] Abbreviations: NI: no interaction, E×C: exposure-confounder interaction, EG: by exposure group.

Table S6: Performance of the complete case analysis (CCA) and six multiple imputation (MI) methods in estimating $\boldsymbol{\theta_1}$ under missingness scenario (i-iii) with 50% exposure prevalence and incomplete outcome.

Outcome scenario	Method	Missingness scenario[a]								
		(i): exposure			(ii): exposure + confounder			(iii): exposure × confounder		
		Bias (%)[b]	EmpSE[c]	Coverage[d]	Bias (%)[b]	EmpSE[c]	Coverage[d]	Bias (%)[b]	EmpSE[c]	Coverage[d]
Strong negative	CCA	-3.418	0.128	95.3	6.072	0.126	95.3	21.803	0.140	92.1
	MI-NI[e]	-3.294	0.129	95.4	6.286	0.126	95.4	21.702	0.142	92.3
	MI-E×C[e]	-1.144	0.128	95.1	-2.098	0.126	95.2	0.197	0.147	94.7
	MI-EG[e]	-1.387	0.129	95.6	-1.908	0.127	95.7	0.162	0.150	95.2
Moderate negative	CCA	-1.380	0.126	95.2	4.616	0.124	95.0	9.160	0.137	95.2
	MI-NI	-1.503	0.127	95.1	4.657	0.125	95.0	9.226	0.137	95.4
	MI-E×C	-0.312	0.127	95.9	0.227	0.126	95.4	-1.986	0.142	96.0
	MI-EG	-0.326	0.128	95.1	0.234	0.126	95.6	-1.859	0.145	95.9
Weak negative	CCA	-0.479	0.131	95.0	1.407	0.129	94.9	4.306	0.139	95.1
	MI-NI	-0.258	0.131	95.0	1.376	0.130	94.6	4.352	0.140	95.1
	MI-E×C	-0.333	0.131	94.9	-0.406	0.131	95.2	0.256	0.145	95.5
	MI-EG	0.135	0.132	95.0	-0.514	0.133	94.6	0.081	0.148	95.6
No interaction	CCA	2.010	0.130	94.7	0.658	0.130	95.5	-0.644	0.143	94.7
	MI-NI	2.092	0.131	94.7	0.717	0.131	95.2	-0.667	0.144	94.5

	MI-E×C	2.140	0.131	95.4	0.249	0.131	95.4	-0.741	0.147	95.0
	MI-EG	2.087	0.132	94.9	0.515	0.132	95.7	-1.193	0.153	94.5
Weak positive	CCA	0.435	0.130	95.0	-2.329	0.128	94.8	-2.037	0.139	95.3
	MI-NI	0.471	0.131	94.6	-2.312	0.129	95.1	-1.873	0.140	96.0
	MI-E×C	0.197	0.131	95.1	-0.928	0.130	95.4	1.598	0.146	94.8
	MI-EG	0.132	0.130	94.7	-0.976	0.130	95.5	1.759	0.148	95.3
Moderate positive	CCA	0.168	0.132	94.9	-3.503	0.127	95.0	-6.100	0.145	94.1
	MI-NI	0.079	0.132	95.3	-3.434	0.128	95.9	-6.187	0.146	93.9
	MI-E×C	-0.675	0.133	94.8	-0.783	0.129	95.1	-0.523	0.149	94.9
	MI-EG	-0.461	0.133	95.0	-0.811	0.129	95.5	-0.301	0.154	94.8
Strong positive	CCA	0.450	0.129	95.4	-2.483	0.126	95.1	-8.715	0.137	95.3
	MI-NI	0.473	0.129	95.6	-2.537	0.128	95.1	-8.684	0.137	95.5
	MI-E×C	-0.445	0.130	95.5	1.283	0.128	94.6	-1.746	0.144	95.7
	MI-EG	-0.470	0.130	95.5	1.218	0.128	94.9	-1.450	0.147	95.4

[a] Missingness scenario (i): missingness only depended on the exposure; missingness scenarios (ii): missingness depended on the exposure and strong confounder; and missingness scenario (iii): missingness depended on the exposure, strong confounder, and their interaction.
[b] Relative bias: the difference between the mean of the θ_1 estimates and the target value of θ_1(0.3), expressed as a percentage of the true value.
[c] Empirical standard error (EmpSE) from 2000 estimated regression coefficients, and Monte-Carlo standard error (MCSE) of EmpSE less than 0.004.
[d] Coverage probability, estimated by the proportion of the 95% confidence intervals that contained the target value of the ACE across the 2000 datasets, and MCSE of coverage less than 0.008.
[e] Abbreviations: NI: no interaction, E×C: exposure-confounder interaction, EG: by exposure group.

Table S7: Performance of the complete case analysis (CCA) and six multiple imputation (MI) methods in estimating θ_1 under missingness scenario (i-iii) with 10% exposure prevalence and incomplete confounders and outcome.

Outcome scenario	Method	Missingness scenario[a]								
		(i): exposure			(ii): exposure + confounder			(iii): exposure × confounder		
		Bias (%)[b]	EmpSE[c]	Coverage[d]	Bias (%)[b]	EmpSE[c]	Coverage[d]	Bias (%)[b]	EmpSE[c]	Coverage[d]
Strong negative	CCA	-0.684	0.141	95.0	14.949	0.147	93.2	29.915	0.160	91.0
	MI-NI[e]	-0.540	0.133	95.0	12.966	0.141	93.3	26.697	0.153	91.2
	MI-E×O[e]	-0.732	0.133	95.3	13.043	0.140	92.9	26.402	0.152	91.3
	MI-E×C[e]	0.065	0.133	95.0	5.134	0.140	94.4	10.214	0.154	94.6
	MI-E×OC[e]	0.337	0.133	95.3	4.749	0.140	94.5	8.980	0.155	94.5
	MI-E×I[e]	0.381	0.135	94.8	4.747	0.141	94.3	8.916	0.156	94.5
	MI-EG[e]	1.366	0.134	94.8	4.695	0.140	94.9	7.243	0.159	94.8

Moderate negative	CCA	0.801	0.139	95.1	6.258	0.141	95.1	14.012	0.156	94.9
	MI-NI	0.532	0.133	94.8	5.374	0.135	94.6	13.154	0.147	94.7
	MI-E×O	0.698	0.133	94.9	5.462	0.135	94.8	13.231	0.148	94.7
	MI-E×C	0.754	0.133	95.0	2.560	0.136	94.7	7.347	0.151	95.8
	MI-E×OC	0.791	0.133	95.0	2.373	0.137	95.3	6.146	0.152	95.2
	MI-E×I	0.731	0.134	95.2	2.219	0.137	94.8	6.367	0.154	95.1
	MI-EG	2.214	0.134	95.1	2.262	0.138	94.9	5.189	0.156	95.3
Weak negative	CCA	0.571	0.136	95.3	2.829	0.145	95.0	7.168	0.154	95.5
	MI-NI	0.589	0.130	95.2	2.397	0.138	94.6	6.724	0.147	95.3
	MI-E×O	0.664	0.130	95.5	2.726	0.139	94.9	6.608	0.147	95.0
	MI-E×C	0.737	0.131	95.4	1.947	0.141	94.8	6.131	0.151	95.5
	MI-E×OC	0.645	0.131	95.2	1.815	0.141	94.9	5.954	0.152	95.3
	MI-E×I	0.818	0.131	95.1	1.856	0.140	94.7	5.724	0.153	95.1
	MI-EG	1.677	0.132	95.3	1.716	0.142	95.0	3.607	0.155	95.6
No interaction	CCA	0.281	0.141	94.5	0.832	0.144	95.0	1.035	0.163	94.6
	MI-NI	0.567	0.134	94.5	0.267	0.137	94.8	2.150	0.152	94.7
	MI-E×O	0.668	0.134	94.7	0.542	0.136	95.2	1.996	0.151	94.9
	MI-E×C	0.228	0.135	94.6	1.339	0.137	94.6	4.584	0.155	94.9
	MI-E×OC	0.463	0.135	94.2	1.594	0.138	94.8	4.858	0.155	95.0
	MI-E×I	0.517	0.135	94.8	1.557	0.138	94.7	5.107	0.156	94.6
	MI-EG	1.443	0.136	94.9	1.351	0.139	95.2	3.311	0.159	95.4
Weak positive	CCA	0.603	0.138	95.1	-1.279	0.145	94.8	-3.531	0.161	94.6
	MI-NI	0.680	0.132	95.2	-1.400	0.137	95.4	-2.568	0.152	93.9
	MI-E×O	0.889	0.132	94.9	-1.600	0.138	95.0	-2.702	0.152	94.3
	MI-E×C	0.401	0.132	95.3	0.505	0.137	95.9	2.369	0.155	94.6
	MI-E×OC	0.523	0.132	95.6	0.457	0.136	95.2	2.593	0.155	94.7
	MI-E×I	0.530	0.133	95.3	0.955	0.137	95.5	2.557	0.156	94.8
	MI-EG	1.270	0.134	95.3	0.240	0.138	95.8	0.920	0.157	94.7
Moderate positive	CCA	0.112	0.140	94.9	-1.238	0.144	94.6	-6.206	0.155	95.9
	MI-NI	0.055	0.133	94.8	-0.669	0.136	94.8	-4.863	0.146	95.9
	MI-E×O	0.098	0.133	94.7	-0.863	0.137	94.9	-4.867	0.147	95.5
	MI-E×C	-0.306	0.134	94.6	1.861	0.137	95.5	1.430	0.149	96.0
	MI-E×OC	-0.497	0.133	95.0	2.035	0.136	95.5	1.540	0.148	95.9
	MI-E×I	-0.380	0.133	94.8	1.881	0.137	95.2	1.927	0.151	96.2
	MI-EG	0.389	0.135	95.2	1.315	0.138	95.8	0.364	0.154	95.9

Strong positive	CCA	0.436	0.139	94.7	-2.760	0.141	95.3	-8.304	0.155	95.5
	MI-NI	0.339	0.134	94.7	-2.787	0.134	95.1	-5.563	0.145	94.9
	MI-E×O	0.465	0.133	94.4	-2.807	0.134	95.0	-5.762	0.145	95.0
	MI-E×C	0.393	0.133	94.7	0.460	0.135	95.2	1.669	0.148	95.8
	MI-E×OC	0.177	0.133	94.3	0.735	0.135	95.1	1.865	0.149	95.3
	MI-E×I	0.175	0.135	94.6	0.529	0.136	95.3	2.046	0.151	95.9
	MI-EG	0.924	0.135	95.1	-0.009	0.138	95.3	0.300	0.153	95.7

[a] Missingness scenario (i): missingness only depended on the exposure; missingness scenarios (ii): missingness depended on the exposure and strong confounder; and missingness scenario (iii): missingness depended on the exposure, strong confounder, and their interaction.

[b] Relative bias: the difference between the mean of the θ_1 estimates and the target value of θ_1(0.3), expressed as a percentage of the true value.

[c] Empirical standard error (EmpSE) from 2000 estimated regression coefficients, and Monte-Carlo standard error (MCSE) of EmpSE less than 0.005.

[d] Coverage probability, estimated by the proportion of the 95% confidence intervals that contained the target value of the ACE across the 2000 datasets, and MCSE of coverage less than 0.008.

[e] Abbreviations: NI: no interaction, E×O: exposure-outcome interaction, E×C: exposure-confounder interaction, E×OC: exposure-confounder and exposure-outcome interactions, E×I: exposure-incomplete variables interactions, EG: by exposure group.

Table S8: Performance of the complete case analysis (CCA) and six multiple imputation (MI) methods in estimating θ_1 under missingness scenario (i-iii) with 30% exposure prevalence and incomplete confounders and outcome.

Outcome scenario	Method	Missingness scenario[a]								
		(i): exposure			(ii): exposure + confounder			(iii): exposure × confounder		
		Bias (%)[b]	EmpSE[c]	Coverage[d]	Bias (%)[b]	EmpSE[c]	Coverage[d]	Bias (%)[b]	EmpSE[c]	Coverage[d]
Strong negative	CCA	-3.379	0.132	94.8	7.454	0.129	94.7	15.487	0.133	93.7
	MI-NI[e]	-3.377	0.126	94.6	6.506	0.124	95.3	14.202	0.128	93.9
	MI-E×O[e]	-3.507	0.126	94.8	6.509	0.123	95.4	14.074	0.128	93.7
	MI-E×C[e]	-2.267	0.126	95.1	1.235	0.124	95.4	5.310	0.129	95.1
	MI-E×OC[e]	-2.118	0.126	95.0	0.913	0.124	95.6	4.684	0.128	95.3
	MI-E×I[e]	-1.997	0.126	94.5	1.079	0.124	95.8	4.771	0.129	95.1
	MI-EG[e]	-1.106	0.126	94.8	0.631	0.125	95.6	3.705	0.129	95.2
Moderate negative	CCA	-2.527	0.128	95.0	3.955	0.129	95.3	6.496	0.133	94.5
	MI-NI	-2.460	0.122	95.7	2.737	0.123	95.4	5.702	0.125	94.6
	MI-E×O	-2.635	0.122	95.7	2.868	0.124	95.2	5.503	0.125	95.0
	MI-E×C	-2.194	0.122	96.1	0.780	0.124	95.5	2.123	0.126	95.3
	MI-E×OC	-2.078	0.122	95.7	0.806	0.124	95.6	1.781	0.127	95.0
	MI-E×I	-1.848	0.122	95.7	0.904	0.124	95.6	1.902	0.127	94.6

	MI-EG	-1.346	0.123	95.6	0.125	0.124	95.4	0.461	0.127	94.7
Weak negative	CCA	0.037	0.130	95.3	2.048	0.132	95.2	3.768	0.133	94.6
	MI-NI	-0.264	0.124	95.7	1.062	0.125	95.1	4.072	0.128	94.8
	MI-E×O	-0.045	0.124	95.3	1.284	0.126	94.9	3.793	0.128	94.6
	MI-E×C	-0.147	0.123	95.6	1.216	0.126	95.3	3.673	0.129	94.6
	MI-E×OC	-0.073	0.124	95.4	0.979	0.126	95.2	3.677	0.130	95.0
	MI-E×I	-0.058	0.124	95.3	1.257	0.126	94.9	3.742	0.130	94.9
	MI-EG	0.537	0.124	95.3	0.616	0.126	94.8	2.381	0.131	94.5
No interaction	CCA	-0.375	0.129	95.5	-0.199	0.135	93.8	-0.942	0.137	94.4
	MI-NI	0.226	0.126	94.9	-0.500	0.128	94.2	0.244	0.131	94.5
	MI-E×O	0.270	0.125	95.0	-0.509	0.129	93.7	0.322	0.131	94.4
	MI-E×C	0.022	0.126	94.6	0.495	0.128	94.5	1.882	0.132	94.3
	MI-E×OC	-0.163	0.126	94.8	0.595	0.128	94.3	2.139	0.132	94.7
	MI-E×I	-0.097	0.126	95.0	0.618	0.129	94.6	2.258	0.132	94.1
	MI-EG	0.435	0.126	94.7	-0.125	0.129	94.4	0.650	0.132	94.5
Weak positive	CCA	2.403	0.129	95.2	-0.313	0.130	95.2	-0.179	0.134	95.1
	MI-NI	2.730	0.125	94.5	0.072	0.127	93.9	1.255	0.129	94.9
	MI-E×O	2.609	0.125	95.0	0.232	0.127	94.2	1.329	0.129	95.0
	MI-E×C	2.275	0.126	94.8	1.978	0.128	94.0	4.801	0.131	94.6
	MI-E×OC	2.015	0.126	94.7	2.011	0.128	94.3	4.853	0.131	94.4
	MI-E×I	2.255	0.126	95.1	1.954	0.128	94.1	5.069	0.132	94.5
	MI-EG	2.717	0.125	94.8	1.225	0.128	94.4	3.154	0.132	94.2
Moderate positive	CCA	-0.574	0.133	94.6	-4.209	0.130	94.8	-5.025	0.133	94.9
	MI-NI	-0.475	0.129	93.9	-4.227	0.126	94.6	-3.719	0.129	95.0
	MI-E×O	-0.425	0.128	94.1	-4.245	0.126	94.1	-3.723	0.129	94.8
	MI-E×C	-1.226	0.128	94.1	-1.799	0.127	94.9	0.878	0.130	94.4
	MI-E×OC	-1.151	0.129	94.0	-1.571	0.127	95.0	0.900	0.130	94.7
	MI-E×I	-1.214	0.129	94.1	-1.673	0.127	94.9	0.738	0.130	94.9
	MI-EG	-0.600	0.130	93.8	-2.218	0.128	95.2	-0.773	0.131	95.1
Strong positive	CCA	0.011	0.127	95.6	-3.813	0.127	95.6	-4.842	0.130	95.2
	MI-NI	0.048	0.121	96.0	-3.625	0.121	95.8	-3.531	0.126	95.1
	MI-E×O	-0.027	0.121	96.1	-3.727	0.121	95.9	-3.444	0.126	95.0
	MI-E×C	-0.863	0.122	95.8	-1.102	0.121	95.6	1.721	0.127	95.0
	MI-E×OC	-0.626	0.121	95.6	-0.767	0.122	95.9	1.735	0.128	95.1
	MI-E×I	-0.719	0.122	96.1	-0.865	0.122	95.8	2.060	0.128	94.9

	MI-EG	-0.373	0.122	96.0	-1.667	0.121	95.9	0.469	0.129	95.2

[a] Missingness scenario (i): missingness only depended on the exposure; missingness scenarios (ii): missingness depended on the exposure and strong confounder; and missingness scenario (iii): missingness depended on the exposure, strong confounder, and their interaction.
[b] Relative bias: the difference between the mean of the θ_1 estimates and the target value of θ_1(0.3), expressed as a percentage of the true value.
[c] Empirical standard error (EmpSE) from 2000 estimated regression coefficients, and Monte-Carlo standard error (MCSE) of EmpSE less than 0.004.
[d] Coverage probability, estimated by the proportion of the 95% confidence intervals that contained the target value of the ACE across the 2000 datasets, and MCSE of coverage less than 0.007.
[e] Abbreviations: NI: no interaction, E×O: exposure-outcome interaction, E×C: exposure-confounder interaction, E×OC: exposure-confounder and exposure-outcome interactions, E×I: exposure-incomplete variables interactions, EG: by exposure group.

Table S9: Performance of the complete case analysis (CCA) and six multiple imputation (MI) methods in estimating θ_1 under missingness scenario (i-iii) with 50% exposure prevalence and incomplete confounders and outcome.

Outcome scenario	Method	Missingness scenario[a]								
		(i): exposure			(ii): exposure + confounder			(iii): exposure × confounder		
		Bias (%)[b]	EmpSE[c]	Coverage[d]	Bias (%)[b]	EmpSE[c]	Coverage[d]	Bias (%)[b]	EmpSE[c]	Coverage[d]
Strong negative	CCA	-3.655	0.127	95.2	4.305	0.127	95.0	7.181	0.123	95.1
	MI-NI[e]	-3.779	0.121	95.7	3.793	0.120	94.9	6.719	0.116	95.4
	MI-E×O[e]	-3.445	0.121	95.5	3.893	0.120	95.0	6.911	0.116	95.4
	MI-E×C[e]	-3.085	0.121	95.5	0.695	0.120	95.4	2.303	0.117	95.9
	MI-E×OC[e]	-3.037	0.120	95.9	0.724	0.119	95.2	1.890	0.116	95.7
	MI-E×I[e]	-2.783	0.120	95.6	0.592	0.120	95.5	2.212	0.117	95.6
	MI-EG[e]	-2.051	0.120	96.0	-0.098	0.120	95.4	1.271	0.116	96.1
Moderate negative	CCA	-1.311	0.124	95.5	2.552	0.122	95.2	4.742	0.121	95.6
	MI-NI	-1.157	0.119	95.2	2.061	0.118	95.6	4.589	0.116	95.6
	MI-E×O	-0.921	0.119	95.5	2.128	0.117	95.4	4.553	0.115	95.6
	MI-E×C	-0.795	0.119	95.7	0.799	0.118	95.6	2.672	0.116	95.4
	MI-E×OC	-0.792	0.119	95.5	0.679	0.117	95.4	2.560	0.117	95.3
	MI-E×I	-0.705	0.119	95.4	0.730	0.117	95.5	2.613	0.117	95.6
	MI-EG	0.004	0.119	95.0	0.194	0.117	95.5	1.789	0.116	95.5
Weak negative	CCA	0.018	0.123	95.8	0.781	0.126	94.8	2.522	0.125	95.2
	MI-NI	0.024	0.118	95.3	0.751	0.121	94.7	2.978	0.121	95.1
	MI-E×O	0.054	0.118	95.4	0.864	0.122	94.4	2.980	0.120	94.9
	MI-E×C	-0.208	0.118	95.5	0.668	0.122	95.1	2.813	0.120	95.7
	MI-E×OC	-0.048	0.118	95.7	0.581	0.121	94.7	2.894	0.120	95.2

	MI-E×I	0.024	0.118	95.7	0.657	0.121	95.0	2.865	0.121	95.0
	MI-EG	0.619	0.118	95.3	0.073	0.122	95.1	1.961	0.121	95.0
No interaction	CCA	0.332	0.129	94.4	1.196	0.128	95.1	0.431	0.129	94.3
	MI-NI	0.808	0.123	94.8	1.224	0.123	94.8	1.139	0.125	94.6
	MI-E×O	0.881	0.123	94.7	1.281	0.123	94.5	1.247	0.125	94.8
	MI-E×C	0.423	0.123	95.0	2.002	0.123	95.3	2.404	0.126	94.4
	MI-E×OC	0.547	0.123	94.8	2.084	0.123	95.1	2.466	0.125	94.6
	MI-E×I	0.452	0.123	95.1	2.142	0.123	95.2	2.446	0.126	94.4
	MI-EG	1.073	0.123	94.8	1.308	0.123	94.7	1.376	0.126	94.8
Weak positive	CCA	-0.071	0.125	94.4	-0.398	0.127	94.5	-1.905	0.125	95.3
	MI-NI	0.337	0.120	94.9	-0.341	0.121	95.0	-0.534	0.120	95.0
	MI-E×O	0.490	0.120	95.5	-0.346	0.121	94.9	-0.563	0.119	94.8
	MI-E×C	-0.014	0.120	95.2	1.248	0.121	94.7	1.776	0.120	95.5
	MI-E×OC	-0.193	0.120	95.1	1.194	0.121	94.8	1.823	0.120	95.5
	MI-E×I	-0.082	0.120	95.3	1.285	0.121	94.7	1.821	0.120	95.2
	MI-EG	0.275	0.120	95.2	0.659	0.121	94.9	0.736	0.119	94.9
Moderate positive	CCA	-0.494	0.129	94.9	-2.290	0.127	94.7	-4.185	0.127	94.7
	MI-NI	-0.232	0.124	95.0	-2.351	0.122	94.7	-3.151	0.122	94.7
	MI-E×O	-0.326	0.125	94.6	-2.395	0.122	94.7	-3.170	0.121	95.1
	MI-E×C	-0.990	0.125	94.8	-0.237	0.122	94.6	-0.037	0.121	94.7
	MI-E×OC	-1.066	0.124	95.2	-0.004	0.122	94.9	0.146	0.122	94.9
	MI-E×I	-0.928	0.125	94.9	-0.073	0.122	94.9	0.369	0.121	95.2
	MI-EG	-0.682	0.124	94.6	-0.917	0.122	94.6	-0.975	0.121	95.1
Strong positive	CCA	1.055	0.128	94.2	-2.364	0.125	94.5	-3.227	0.124	95.1
	MI-NI	1.375	0.121	94.7	-2.327	0.121	94.4	-2.181	0.119	95.0
	MI-E×O	1.442	0.121	94.2	-2.487	0.121	94.8	-2.232	0.119	95.3
	MI-E×C	0.586	0.122	94.6	0.421	0.122	94.7	1.577	0.120	95.4
	MI-E×OC	0.439	0.121	94.9	0.409	0.121	94.9	1.721	0.120	94.8
	MI-E×I	0.686	0.122	94.8	0.225	0.121	94.8	1.657	0.120	95.0
	MI-EG	1.202	0.121	94.8	-0.274	0.121	94.9	0.672	0.120	95.0

[a] Missingness scenario (i): missingness only depended on the exposure; missingness scenarios (ii): missingness depended on the exposure and strong confounder; and missingness scenario (iii): missingness depended on the exposure, strong confounder, and their interaction.

[b] Relative bias: the difference between the mean of the θ_1 estimates and the target value of $\theta_1(0.3)$, expressed as a percentage of the true value.

[c] Empirical standard error (EmpSE) from 2000 estimated regression coefficients, and Monte-Carlo standard error (MCSE) of EmpSE less than 0.004.

[d] Coverage probability, estimated by the proportion of the 95% confidence intervals that contained the target value of the ACE across the 2000 datasets, and MCSE of coverage less than 0.007.
[e] Abbreviations: NI: no interaction, E×O: exposure-outcome interaction, E×C: exposure-confounder interaction, E×OC: exposure-confounder and exposure-outcome interactions, E×I: exposure-incomplete variables interactions, EG: by exposure group.

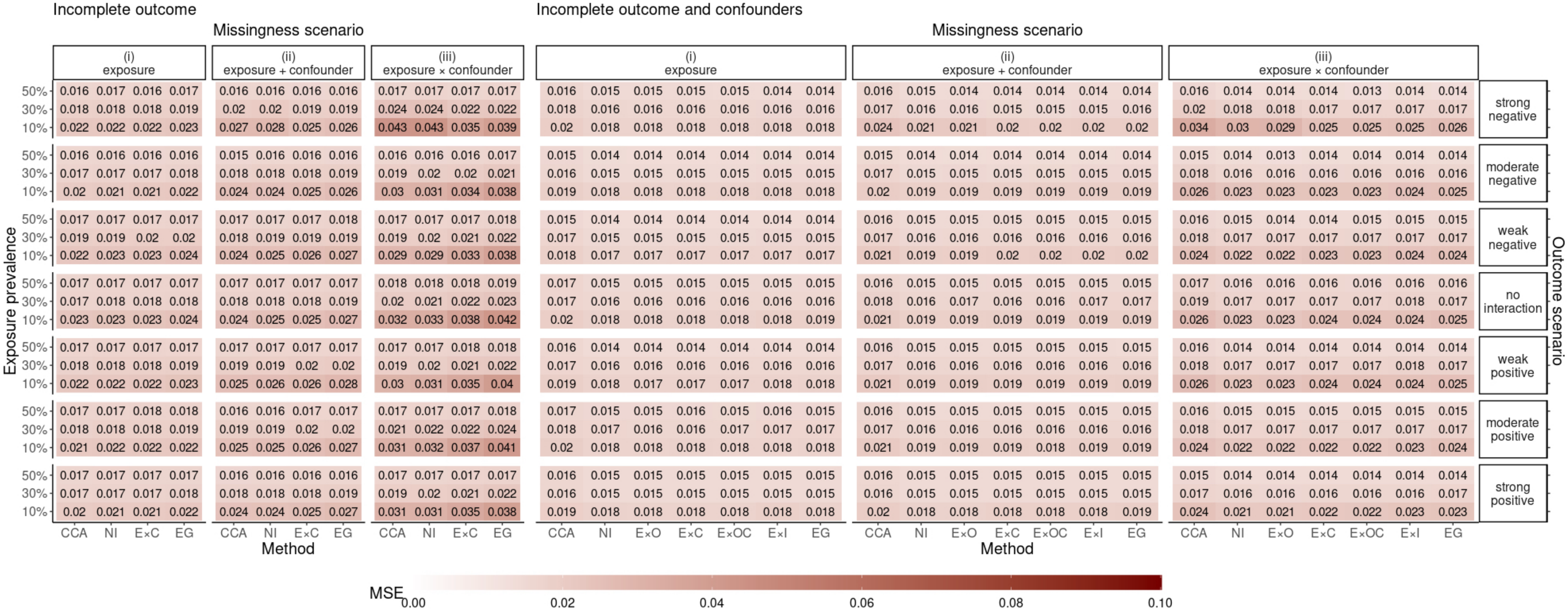

Figure S1: The mean squared error (MSE) of the complete case analysis (CCA) and six multiple imputation (MI) methods in estimating θ_1 across all missingness, outcome and exposure-prevalence scenarios.

[a] Missingness scenario (i): missingness only depended on the exposure; missingness scenarios (ii): missingness depended on the exposure and strong confounder; and missingness scenario (iii): missingness depended on the exposure, strong confounder, and their interaction.

[b] Mean squared error (MSE): the sum of the squared bias and variance of the 2,000 estimates

[c] Abbreviations: NI: no interaction, E×O: exposure-outcome interaction, E×C: exposure-confounder interaction, E×OC: exposure-confounder and exposure-outcome interactions, E×I: exposure-incomplete variables interactions, EG: by exposure group.

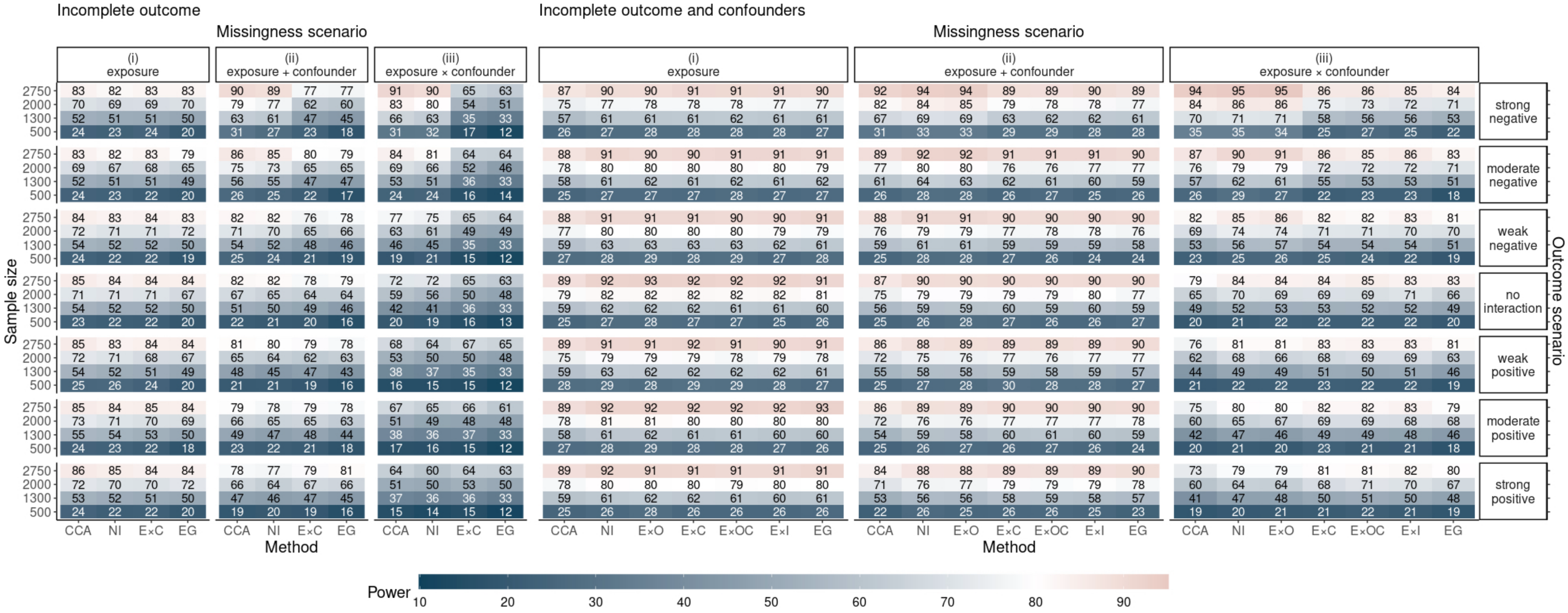

Figure S2: Power (%) of the complete case analysis (CCA) and six multiple imputation (MI) methods in estimating θ_1 for different sample sizes across missingness and outcome scenarios with 10% exposure prevalence.

[a] Missingness scenario (i): missingness only depended on the exposure; missingness scenarios (ii): missingness depended on the exposure and strong confounder; and missingness scenario (iii): missingness depended on the exposure, strong confounder, and their interaction.

[b] Abbreviations: NI: no interaction, E×O: exposure-outcome interaction, E×C: exposure-confounder interaction, E×OC: exposure-confounder and exposure-outcome interactions, E×I: exposure-incomplete variables interactions, EG: by exposure group.
